# Formulation and *In Vitro* Evaluation of Rifampicin Loaded Porous Microspheres

**DOI:** 10.3797/scipharm.0910-09

**Published:** 2010-04-18

**Authors:** Satish Balakrishna Bhise, Avinash Bhanudas More, Rajkumar Malayandi

**Affiliations:** Biopharmaceutical Research Group, Dpt. of Biopharmaceutics, Govt. College of Pharmacy, Karad, India

**Keywords:** Tuberculosis, Rifampicin, Porous micro-spheres, Biopharmaceutics

## Abstract

Rifampicin (RIF) is a major component in fixed dose combination therapy for the treatment of tuberculosis. RIF has low solubility and high permeability with high dose and hence it is classified as class II drug in Biopharmaceutical Classification System (BCS). RIF has poor and variable bioavailability because of its poor solubility, acid decomposition and, drug and food interaction. The present investigation was aimed to develop RIF loaded porous microspheres as a controlled release dosage form. Eudragit based porous microspheres of RIF were prepared by emulsion solvent diffusion method. Prepared porous microspheres were evaluated for its entrapment efficacy, morphology, thermal behavior, crystalline nature, in-vitro drug release and stability in simulated gastric fluid. The entrapment efficacy of drug loaded microspheres was found to be in the range of 19.04–74.57%. Surface morphology revealed the porous and spherical structure of microspheres. Differential scanning calorimetric studies confirmed that formulation process altered the crystalline nature of RIF. In vitro drug release studies indicated that drug to polymer ratio of 2:1 showed more than 85% drug release over the period of 3 h. Stability studies in simulated gastric fluid (SGF) indicated that low relative decomposition of 18.5% was achieved with high drug to low polymer ratio of 1:4. The results obtained from the present investigation concluded that RIF loaded porous microspheres are suitable for developing oral controlled release dosage form of RIF that can prevent acid decomposition and provide better biopharmaceutical properties. Further more the microspheres can be evaluated for preventing the interaction with isoniazid, other drugs and foodstuffs.

## Introduction

Tuberculosis (TB) is one of the major infectious diseases worldwide and its incidence is increasing particularly in association with AIDS pandemic [1]. The four major drugs rifampicin (RIF), isoniazid (INH), pyrazinamide (Z) and ethambutol (E) are used widely for the treatment of TB [2]. Among these, INH, Z and E belong to the class I (highly soluble/highly permeable) of biopharmaceutical classification system whereas RIF is a class II drug (low soluble/highly permeable), which has high permeability and low solubility [3]. Due to the low solubility, RIF (solubility 2.5 mg/ml, log *p* 1.086, p*K*a 4.96±0.7) has poor bioavailability in both fixed and single dose formulations [4]. The reason for low bioavailability of RIF are changes in the crystalline nature, interaction with excipients, degradation in gastro-intestinal tract, variability in absorption and metabolism, its pH dependent solubility, etc. [5, 6]. Bioavailability problem of RIF is cause for the poor therapeutic outcomes including failure of therapy and drug resistance. Chemotherapy of TB is complicated by the need of multi-drug regimens given over long periods, patient non-compliance and the development of multi-drug resistant strains (MDR) [4]. Poor patient compliance is another single most common reason for failure of TB therapy [7]. Development of carrier delivery systems that release drugs in a sustained manner at therapeutic concentration over a period of time can ensure patient compliance in terms of reducing dosing frequency, and may also minimize the risk of emergence of drug resistant mutants and potential toxicity [8, 9]. Various carrier systems such as liposomes and microspheres have been developed for the sustained delivery of anti tubercular drugs in mice with better chemotherapeutic efficacy [10, 11]. However, these formulations have to be injected either subcutaneous or intravenous route, which in general is not acceptable. Hence, there is a continuous need for development of an oral drug delivery system that is convenient for patients. Due to the low solubility, acid decomposition and absorption window (RIF has better absorption in stomach due to acidic pH), only few attempts are made to develop the oral controlled release formulations for RIF [12]. The present investigation is aimed to develop the controlled release porous microspheres of RIF that can prevent not only acid decomposition but also controlled release profile of RIF for prolonged period of time.

## Results and Discussion

### Preparation of microspheres

Porous microspheres were prepared by emulsion solvent diffusion method. Various amounts of RIF ranging from 0.1 g to 1.0 g were used for preparation of microspheres. Drug content of above 0.3 g was given poor spherical structure and hence it was not selected for further optimization. Operation temperature ranging from 30–50°C was evaluated during the formulation development. At 30 °C, diffusion rate of ethanol was found to be more than that of evaporation of dichloromethane and hence it was resulted with a sticky mass and formation of microspheres with poor spherical shape. In contrast to that at high temperature (of 50°C), evaporation of DCM was faster than the diffusion of ethanol. This led to improper formation of microspheres. The research results revealed that 40°C found to be the optimal operation temperature for formation of porous microspheres. Concentration of Eudragit was an important parameter, which determined the stability of microspheres during formulation. Drug to polymer ratio ranging from 1:1 to 1:4 provided stable and porous microspheres.

### Entrapment efficacy

Entrapment efficacy of drug-loaded microspheres was found to be in the range of 19.04–74.57% (table 1). Entrapment efficacy was dependent on the composition of drug as well as Eudragit. Low content of Eudragit and high composition of drug in formulation resulted with low entrapment of drug in microsphere formulations. Drug to Eudragit ratio of 1:4 yielded maximum entrapment efficacy of 74.57 %, whereas minimum entrapment efficacy of 19.04% was observed with 3:1 ratio of drug to Eudragit. However, RIF being a low solubility drug with high dose required low concentration of polymer and/or excipients in dosage form for better formulation development. Further research is required to improve the biopharmaceutical properties of RIF using porous microspheres as a carrier.

### Scanning Electron Microscopy

**Fig. 1.**
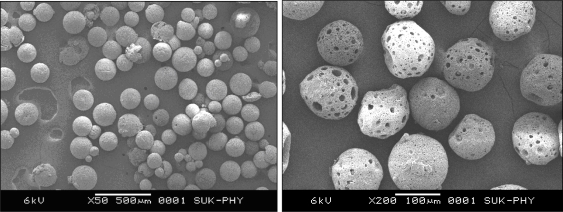
SEM image of Porous microspheres

**Fig. 2.**
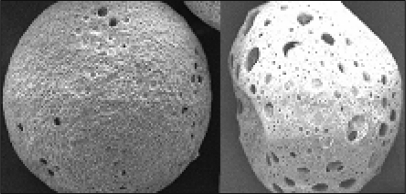
Effect of Eudragit on porosity of microspheres (left: drug to polymer ratio of 1:4; right: drug to polymer ratio of 1:3)

The results obtained from SEM studies confirmed the porous and spherical structure of microspheres (Fig. 1). Moreover, morphology of microspheres revealed that degree of porosity of microspheres were dependent on the composition of Eudragit present in the microspheres. High content of Eudragit lead to loss of porosity. There was no pores appeared in microspheres with above 500 mg of Eudragit in formulation, whereas less than 50 mg content of Eudragit resulted in loss of spherical structure and mechanical strength. The effect of Eudragit on porosity is illustrated in Fig 2. The research is aimed to develop the porous microspheres, which can control the drug release up to 3 h and hence it can prevent the acid decomposition and re-crystallization of RIF in stomach. The results obtained from SAM 3 batch resulted in optimal porosity and good spherical structure.

### Differential Scanning calorimetry

**Fig. 3.**
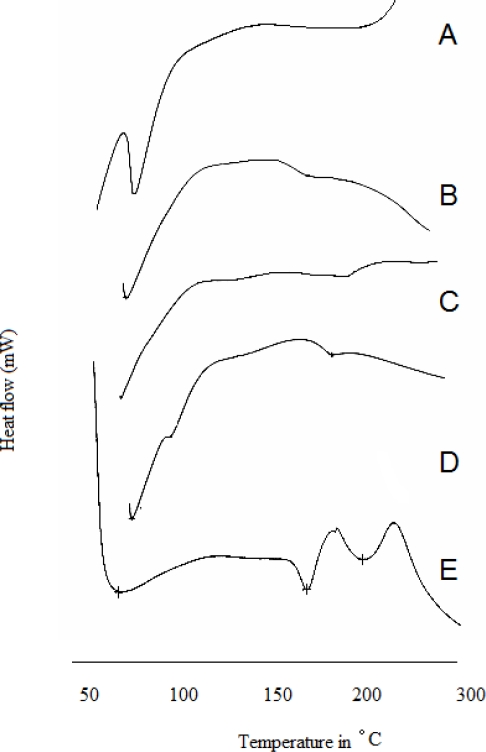
DSC thermogram of RIF, Polymer, physical mixture and microsphere A. GMS ; B. Eudragit ; C. Physical mixture ; D. SAM_4_ microspheres ; E. RIF

RIF exhibits in two polymorphic forms, i.e. form I and form II [13]. It also exists as hydrates and various solvates, which were eventually converted into amorphous form at room temperature or after dissolvation [14]. RIF form II, which is metastable, melts at 189°C and recyrstallize as a form I at 204 °C. RIF form I has lower chemical stability at higher temperature and hence it decompose before melting at 258 °C. Amorphous RIF shows exothermic peak, which started at 196 °C, which indicated the crystallization of form I and subsequent peak at 258 °C represented the decomposition of RIF form I. DSC thermogram of RIF, GSM and Eudragit are shown in Fig 3. DSC thermogram of pure RIF showed endothermic peak at 194.03 °C, which represented the melting of form II and subsequently exothermic peak at 205.62 °C revealed the crystallization of form I. Endothermic peak at 236.07 °C revealed the decomposition form I. Thermal behavior of RIF obtained from DSC data confirmed that RIF used in present study exists in form II. DSC thermogram of RIF microspheres exhibited small endothermic at 246.29 °C, which represented the form I and, confirmed the phase transition (form II to form I) of RIF during formulation development. Small exothermic peak intensity of form I from DSC thermogram of RIF microspheres indicated that the major portion of RIF in microspheres exists in amorphous form. Solid-state stability of RIF is the major problem for poor bioavailability of RIF in dosage forms. The study revealed that emulsion diffusion method for preparation of RIF microspheres altered the crystalline nature of RIF. Solubility and dissolution studies in reported literatures revealed that there was no statistically significant difference in solubility and dissolution between two forms of RIF. Moreover, form I is also present in commercial dosage form and hence there was no impact of change in polymorphic form during formulation development on solubility and dissolution. However, chemical stability of RIF in dosage form is an important critical issue, which can affect the biopharmaceutical quality of dosage form. Emulsion solvent diffusion method for preparation of RIF microspheres was found to be ideal and no impact of process on chemical stability of RIF.

### Powder X ray diffraction

**Fig. 4.**
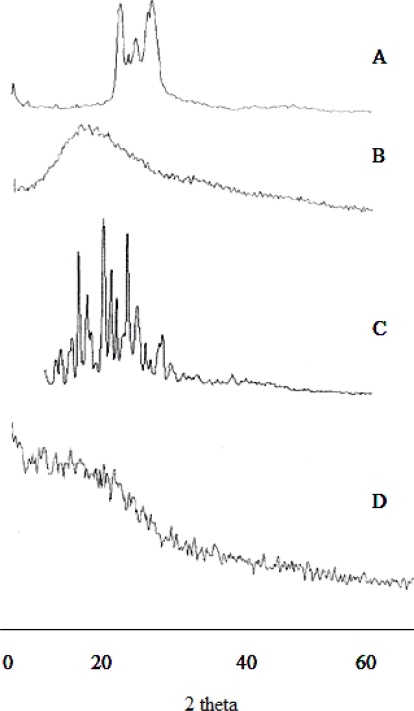
Powder X ray diffractogram of RIF, Polymer and microsphere A. GMS ; B. Eudragit ; C. RIF; D. SAM_4_ microspheres

PXRD pattern of RIF, excipients and microspheres formulation are illustrated in Fig 4. The peaks at 2 Θ of 9.86 and 2 Θ of 11.84 obtained from RIF confirmed that form II crystals of RIF. The hundred percent intensity at 2 Θ of 11.84 confirmed that the presence of form II crystals in pure drug. The absence of peak at 2 Θ of 13.65 and 14.95 indicated that the RIF form I crystals were absent in the pure drug [15]. However, there was no attempt made to quantify the form II present in the RIF. PXRD pattern of microspheres revealed the very weak peak intensity at 2 Θ of 13.17 and 15.12 and confirmed that the phase transition of form II to form I during the formulation development. This result also revealed the major portion of drug exists in the amorphous form. The results obtained from PXRD studies were further confirmed by DSC thermogram.

### In vitro dissolution

**Fig. 5.**
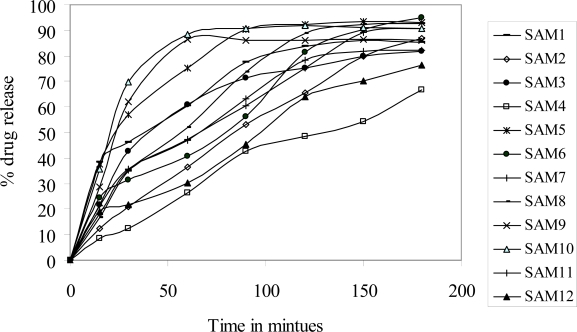
Dissolution profile of RIF porous microspheres

In vitro dissolution studies were performed in phosphate buffer 6.8 as a dissolution media in order to avoid acid decomposition of RIF in presence of HCl. The rate and extent of decomposition of RIF in HCl may affect the quantification of release kinetics of RIF in dissolution medium. Microspheres containing 50 mg equivalent of RIF were subjected to this study to maintain the sink condition throughout the study. Dissolution studies were carried out over the period of 3 h, which mimic the gastric emptying time. The results obtained from dissolution studies are graphically represented in fig 5. All the prepared microspheres showed controlled release profile. T_50%_ for all the formulation were found to be less than 3 h. Drug to Eudragit ratio of 2:1 provided more than 85% release within 3 h. The low release profile of 66.85 % was achieved with formulation containing drug to Eudragit ratio of 1: 4. High drug to low Eudragit ratio contained microspheres showed burst release rather than controlled release profile. Drug to Eudragit ratio of 4:1 to 2: 1 contained microspheres has showed immediate release rather than controlled release profile. The in vitro drug dissolution studies revealed that high drug to low Eudragit ratio contained microspheres can be used to prepare immediate release formulations, whereas low drug to high Eudragit ratio containing microspheres could be used to prepare modified release dosage form of RIF for improving biopharmaceutical characteristics of RIF.

### Stability of RIF in SGF

The purpose of current research work was not only to control the release profile of RIF, but also improve the stability of RIF in presence of gastric fluid. In order to find out the stability of RIF in stomach, SGF without pepsin [16] was used as a medium for determination of relative decomposition of RIF over the period of 3 h. Decomposition of RIF in micro-spheres was less than that of pure drug (Table 1). The low relative decomposition of 18.5 % was achieved with low drug to high polymer ratio of 1:4. Moreover, drug decomposition in microspheres was dependent on the composition of microspheres. Low drug with high Eudragit polymer concentration showed maximum stability of RIF in SGF. Maximum relative decomposition of 34.3 % was obtained from drug to polymer ratio of 1:1 and 4:3. Stability studies of RIF revealed that microspheres reduced the drug decomposition and hence improved the stability of drug in stomach.

## Conclusion

From the present research work, it can be concluded that the formulation of RIF microspheres is a novel approach to improve the stability of RIF and also control the release profile of the drug up to 3 h. Emulsion solvent diffusion method was found to be effective for preparation spherical and porous microspheres. Drug to Eudragit ratio of 1:1 to 1:4 has provided not only spherical porous microspheres but also improved stability and delay rate of drug release. The stability of RIF in in-vitro environments (during formulation and in SGF) was achieved with RIF porous microspheres. DSC studies confirmed that the major portion of drug was in amorphous form. However, small trace of form I found in DSC thermogram demonstrated the phase transition (form II to form I) of RIF during microspheres formation. The present study was aimed to develop the RIF microspheres that can prevent decomposition of RIF in presence of gastric fluid. However, these formulations could be a part of Fixed Dose Combination, which can prevent the interaction between RIF and INH. Further in vitro and in vivo studies are required to explore the clinical applicability of RIF microspheres particularly in presence of INH.

## Experimental

### Materials

RIF was gifted from Lupin Research Park, Pune. Glyceryl monostearate was purchased from Loba chemi, Mumbai. Eudragit RLPO was gifted by Alembic Ltd., Baroda. Ethanol AR and Dichloromethane (HPLC grade) were purchased from Changshu Yangyuan chemical, China and Loba Chemie, Mumbai, India, respectively. All other chemicals used were of analytical grade or HPLC grade.

### Methods

#### HPLC Analysis of RIF

The HPLC system consisted of PU – 2080 plus intelligent system pump, a sampler with 20μl loop, a UV detector UV– 2075 plus intelligent set at 254 nm and an interface LC-Net II/ADC, all from Jasco (Tokyo, Japan). Samples from dissolution and stability experiments were analyzed by a reverse-phase HPLC assay [17]. Detection of RIF was accomplished by ultraviolet absorption (254 nm). The reverse-phase column was a Whatman® C18-RP, 4.6mm×250mm column packed with 5 μ paticles. The mobile phase consisted of phosphate buffer (0.5mM): acetonitrile (65:35). The flow rate was 1 ml/min and the injection volume was 20 μl. The retention time of RIF was 8 min. Quantification was done by use of external standards. The calibration curve was linear in the range 1.5–100 μg/ml. Inter-day precision of the HPLC analysis was estimated by calculating the standard deviation of measurements of the external standards on separate days. The coefficient of variation was then calculated by dividing the standard deviation of the measurements with the mean value. Calculations were made on the highest and lowest concentration of external standards used in the HPLC analysis.

#### Preparation of microspheres

Porous microspheres were prepared by the emulsion solvent diffusion method established by Kawashima [18] as follows: a drug (0.1–0.3 g), Eudragit® RLPO (0.1–0.4 g) and monostearin (GMS) (50 mg) were dissolved or dispersed in a mixture of dichloromethane (8 ml) and ethanol (2 ml) at room temperature. The resulting solution was poured into an aqueous solution of polyvinyl alcohol (0.25w/v%, 200 ml) at 30, 40 and 50°C. The resultant emulsion was stirred at 500 rpm employing a propeller type agitator for 1 h. Subsequently; the resulting microspheres were collected and dried overnight at 40°C. Composition of optimized formulations is listed in table 1.

### Pharmaceutical Characterization of porous microspheres

#### Encapsulation Efficacy

The RIF content in the microspheres was determined by pulverizing the RIF-loaded microspheres (10 mg) followed by immersing them in 100 ml methanol with agitating at room temperature for 10 min. After filtration through a 0.45 μm membrane filter (Millipore), the drug concentration was determined HPLC method. All samples were analyzed in triplicate and the encapsulation efficiency (EE) was calculated according to the following equation:

$$\%\text{Encapsulation}\;\text{Efficacy}=\frac{\text{WA}}{\text{WT}}\cdot 100$$

WA: actual drug content;
WT: theoretical drug content.

#### Scanning Electron Microscopy

The surface topography of the microparticles was examined using optical microcopy and scanning electron microscope (Jeol, JSM-5200, Japan, 15 KV). Samples were coated with gold film under vacuum using a sputter coater (SPI Sputter™ Coating Unit, SPI Supplies, Division of Structure Probe, Inc., PA, USA) and then investigated.

#### DSC analysis

Differential scanning calorimetric (DSC) analyses of the RIF and microspheres were carried out by using differential scanning calorimeter equipped with computer analyzer (Shimadzu TA–60 differential scanning calorimeter, Shimadzu Corporation, Kyoto, Japan). Samples (of 3–7 mg) were heated under nitrogen atmosphere on an aluminum pan at a heating rate of 10 °C/min over the temperature range of 50–300 °C.

#### Powder X-ray diffraction studies

Powder X-ray diffraction (PXRD) patterns were traced employing X-ray diffractometer (Philips PW 1729, Analytical XRD, Holland) for the samples using Ni filtered CuK(α) radiation (intensity ratio(α_1_/ α_2_): 0.500), a voltage of 40 KV, a current of 30 mA and receiving slit of 0.2 inches. The samples were analyzed over 2Θ range of 5.010–39.990° with scanning step size of 0.020 ° (2Θ) and scan step time of one second.

#### In Vitro drug dissolution studies

The drug release rate from porous floating microspheres was determined using USP XXIII basket type dissolution apparatus (Lab India Disso 2000, Lab India Ltd., Mumbai). A weighed amount of porous microspheres equivalent to 50 mg drug was filled into a capsule (# 3) and placed in the basket. Phosphate buffer pH 6.8 was used as the dissolution medium and maintained at 37 ± °C at a rotation speed of 100 rpm. Perfect sink conditions prevailed during the drug release studies. Five ml sample was withdrawn at each 15 min, 30 min, 60 min, 90 min, 120 min, 150 min and 180 min interval, passed through a 0.45 μm membrane filter (Millipore), and analyzed by HPLC method to determine the concentration of drug present in the dissolution medium. The initial volume of the dissolution fluid was maintained by adding 5 ml of fresh dissolution fluid after each withdrawal. All experiments were conducted in triplicate.

## Stability of Rifampicin in simulated gastric fluids

Stability of RIF in microspheres was monitored for 3 h using simulated gastrointestinal fluid (without pepsin) as solvent. In brief, formulation contained 10 mg equivalent of RIF were dispersed in 100 ml of SGF (S) and incubated in digital shaker with 100 rpm at 37 ºC for 3 h (Digital incubator shaker, Samruth Electronics, New Delhi). The resultant solution and/or suspensions were filtered through 0.2 filters and analyzed by HPLC for its stability. The total area of detector response other than RIF was considered as extent of decomposition. Percentage relative decomposition (%RD) was calculated using pure RIF as a standard.

$$\%\text{RD}=\frac{{\text{TPOR}}_{F}}{{\text{TPOR}}_{P}}\cdot 100$$

TPOR_F_: Total area of peak other than RIF peak in formulation
TPOR_p_: Total area of peak other than RIF peak in Pure drug

## Figures and Tables

**Tab. 1.** Composition and Pharmaceutical characterization of RIF porous microspheres

**Batch Code**	**Composition in mg**			**T 50% (min)**	% EE ± SD*	% RD ± SD*
	**RIF**	EUD**	**GMS**			
SAM 1	100	100	50	30	56.12 ± 1.34	34.5 ± 3.7
SAM 2	100	200	50	90	63.99 ± 1.80	31.3 ± 2.9
SAM 3	100	300	50	60	67.43 ± 3.08	23.3 ± 5.5
SAM 4	100	400	50	120	74.57 ± 1.15	18.5 ± 1.8
SAM 5	200	100	50	30	35.72 ± 2.01	45.9 ± 6.0
SAM 6	200	200	50	60	41.40 ± 2.88	29.5 ± 1.4
SAM 7	200	300	50	60	49.68 ± 1.96	25.0 ± 2.9
SAM 8	200	400	50	30	60.61 ± 0.75	25.7 ± 4.0
SAM 9	300	100	50	30	19.04 ± 3.12	34.3 ± 5.6
SAM 10	300	200	50	30	23.83 ± 1.26	31.4 ± 3.4
SAM 11	300	300	50	60	42.77 ± 2.14	16.8 ± 1.9
SAM 12	300	400	50	900	58.29 ± 2.76	11.4 ± 3.7

* standard deviation (n=3),

** Eudragit
